# The Opening of Pandora's Box: An Emerging Role of Long Noncoding RNA in Viral Infections

**DOI:** 10.3389/fimmu.2018.03138

**Published:** 2019-01-25

**Authors:** Pin Wang

**Affiliations:** National Key Laboratory of Medical Immunology and Institute of Immunology, Second Military Medical University, Shanghai, China

**Keywords:** lncRNA, long noncoding RNA, immune response, viral infection, antiviral immunity, RNA-protein interaction

## Abstract

Emerging evidence has proved that long noncoding RNAs (lncRNAs) participate in various physiological and pathological processes. Recent evidence has demonstrated that lncRNAs are crucial regulators of virus infections and antiviral immune responses. Upon viral infections, significant changes take place at the transcriptional level and the majority of the expression modifications occur in lncRNAs from both the host and viral genomes with dynamic regulatory courses. These lncRNAs exert diverse effects. Some are antiviral either through directly inhibiting viral infections or through stimulating antiviral immune responses, while others are pro-viral through directly promoting virus replication or through influencing cellular status, such as suppressing antiviral mechanisms. Consequently, these dynamic regulations lead to disparate pathophysiological outcomes and clinical manifestations. This review will focus on the roles of lncRNAs in viral infection and antiviral responses, summarize expression patterns of both host- and virally derived lncRNAs, describe their acting stages and modes of action, discuss challenges and novel concepts, and propose solutions and perspectives. Research into lncRNA will help identify novel viral infection-related regulators and design preventative and therapeutic strategies against virus-related diseases and immune disorders.

## Introduction

In the RNA world hypothesis, RNA was proposed to be the original form of life, at least the vital compartment of original life, as its spatial structure possesses two major characteristics that biological functional macromolecules required—diversity and flexibility. However, during subsequent process of life development, RNA transferred its role of information storage to DNA that is more stable, and its catalytic activity to protein which has more sophisticated spatial structure, while RNA itself gradually becomes the intermediate between DNA and protein in life organization. RNA only reserved its activity diversity in some fundamental complexes, such as spliceosome, telomerase, ribosome, signal recognition particle (SRP), some metabolic riboswitches, and ribozymes. This is described as the center dogma of modern molecular genetics and was deeply believed by the academic community until the revealing of large portion of noncoding RNA transcript in the latest annotation of genome sequences and interpretation of transcriptome data. We now know that the role of RNA is much further beyond just the message of genome information, it still preserves its ancient diversity and mystery, leaving us to discover.

Facilitated by fast-developing sequencing techniques and bioinformatics, genomes, and transcription profiling in the early Twenty-first century were conducted in human being and other metazoan species, leading to the unexpected observation that while majority of genome is transcribed only small portion is protein coding sequences (approximately 2% in mammalian genome) (1, 2). More detailed annotation and advanced bioinformatics analysis further helped us to reveal various epigenetic elements and genomic origination of these noncoding genes in different cell types and tissues, through projects such as Encyclopedia of DNA Elements (ENCODE) (3) and The Cancer Genome Atlas (TCGA) Research Network (4). The complexity has led us to shift our understanding of genomic information from linear model to modular model, which combines transcription and function of noncoding RNA with DNA regulatory elements, epigenetic modification and spatial origination.

Amid this progress, as a transcriptional class, lncRNAs were first described in the year of 2002 by Okazaki et al. in the study of large-scale sequencing of full-length cDNA libraries in mouse (5). Actually, lncRNA is an arbitrary category definition mainly referring to RNA transcripts with no obvious peptide coding capacity, usually longer than 200 nucleotides to distinguish from short noncoding RNA, such as microRNA, short interfering RNAs (siRNAs), and Piwi-interacting RNAs (piRNAs). Both long and short noncoding RNA compose regulatory RNAs with diverse unknown functions, in contrast to the housekeeping RNAs with certain functions including ribosomal RNA (rRNA), transfer RNA (tRNA), and small nucleolar RNAs (snoRNAs). However, some recent evidences suggest that some tRNA derived RNA fragments also have regulatory function in diverse aspects, such as intergenerational inheritance (6) and viral infection (7).

The number of lncRNA transcripts being identified keeps increasing these years. Up to now, according to a comprehensive bioinformatics analysis using data from 25 independent studies, 58,648 genes were identified as lncRNAs for human being (8). Interestingly, the number of lncRNAs correlates with the developmental complexity of species, at least in all the annotated eukaryotes, with highest lncRNAs amount in primates followed by mouse and scaling down accordingly to yeast (9). While the transcriptional sequence of lncRNAs are less conserved than that of protein coding genes, their promoter sequences and genomic locations are as conserved as coding genes (10, 11), indicating their expression are under tightly regulatory control and evolutional constrains. This is supported by the observation that lncRNA expression profiles are more tissue specific and cell-type selective than that of coding RNA (8), suggesting lncRNAs prefer to perform subtly function in cell-type specific manners, although majority of them are still less characterized.

**Figure 1:**
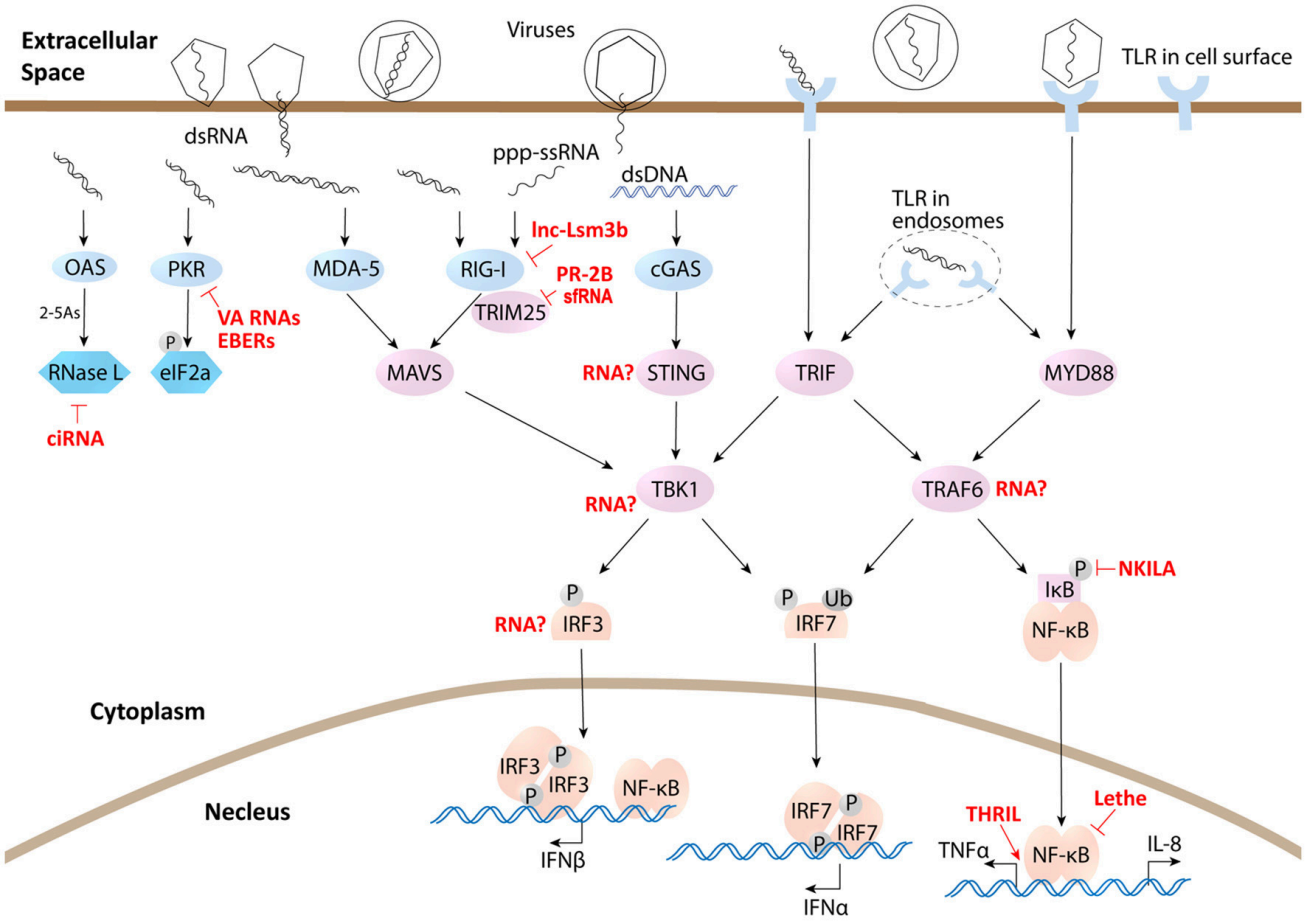
lncRNAs influence immune defense responses through directly interacting with sensors, adaptors, effecters, and transcriptional factors. Light blue represents sensors, cyan represents effecters, light purple represents signaling adaptors, and light brown represents transcriptional factors.

Another protagonist of this review is virus. As an anciently derived organization as RNA, virus has some unique connection with RNA molecules. Virus is the only organism on this planet that reserves RNA as the genome and its RNA could be replicated through RNA dependent RNA polymerase and translated into DNA through reverse transcriptase in the life cycle of viral infection, which makes RNA spectrum more diverse in infected cells, including host-derived, virally derived and even some chimeric RNAs. However, host cells have developed mechanisms to distinguish virally derived RNA from its own RNA through pattern recognition receptors (PRRs), such as Toll-like receptors (TLR) and RIG-I-like receptors (RLR). Viral transcribed RNA usually has a 5′ triphosphate uncapped terminal, which could be recognized by host canonical sensor RIG-I (12, 13). Double-stranded RNA (dsRNA) is commonly produced during viral infection as genetic materials or replicating intermediates during virus replication, which will trigger dsRNA sensors in host cells, including canonical sensor MDA5 in the cytoplasm (14) and membrane receptor TLR3 (15) (Figure 1). Interaction between virus and its host has never halted since its very beginning. Being the simplest but efficient obligated parasites, virus have evolved a variety of strategies to manipulate hosts to provide material and energy to complete their life cycle, including duplicating their genome, producing their RNAs and proteins, packaging infective particles and finally releasing to infect other host cells. On the other hand, the host has developed an immune system whose activation launches immune responses to eliminate viral infections. Many viral components or their intermediate, such as their nucleic acids, can activate host immune system when being recognized by host cell PRRs and subsequently trigger downstream cascade signaling transduction (Figure 1). With the help of adaptor proteins, serine/threonine-protein kinase TBK1 phosphorylates IRF3/7 and TRAF6 activates Nuclear factor-κB (NF-κB) signaling, leading to the transcription of type I interferon (IFN) and inflammatory cytokines, along with many noncoding genes (16, 17) (Figure 1). IFN is the most efficient antiviral cytokine. Through JAK/STAT signaling, IFN triggers lots of effecter genes' expression including many noncoding genes, and induces the antiviral status of host cells to defeat invasion. However, uncontrolled antiviral responses and inflammatory status are also detrimental to host cells. So, immune responses must be finely regulated to minimize cytotoxic effect and autoimmunity output, which requires negative feedback regulatory mechanism to control the duration and magnitude of antiviral responses. A large amount of proteins and increasing lncRNAs have been proved to be involved in this regulatory loop. However, during a long time evolution, these regulatory factors are also utilized by some virus to escape host defense in many cases. The competing between host cells and virus has evolved to be a mutual-driving interaction involving more and more regulatory proteins and lncRNAs from both sides. As a research hotspot, interests and studies progress rapidly in recent years. While many reviews have been published on this theme in recent years (9, 18–20), more and more host- or viral- encoded lncRNAs have been characterized and novel action models of lncRNA functions have been revealed. For example, lncRNA directly regulates metabolic activity of the host cells and lncRNA interacts with singling adaptors or sensors to exert functions. This review will focus on these recent advances and cutting-edge technologies in this area to present a comprehensive view of mammalian host- and viral- derived lncRNAs.

## Virally Derived RNAs

The existence of viral noncoding RNAs has already been known for decades (21, 22). In the year of 1971, it was reported in the plant that viroids, as the smallest infectious pathogens known, are composed solely of a short strand of circular, single-stranded RNA which are capable of autonomous replication (23, 24). So viroid has been considered to be the living relics of the hypothetical RNA world. The expression of noncoding RNAs in host cells from animal viruses has also been described years ago. Some of them are very abundant after infection, such as PAN (polyadenylated nuclear RNA) from Kaposi's sarcoma-associated herpesvirus (KSHV) (25), EBER1/2 (Epstein-Barr virus encoded small RNA 1 and 2) from Epstein-Barr virus (26, 27) and VA RNA (virus-associated RNA) from adenovirus (28). Aside from duplication by RNA-dependent RNA polymerase, some viral RNAs are transcribed by host polymerase III, such as VA RNAs and EBER1/2, while some are transcribed by polymerase II and polyadenylated, such as PAN RNA (Table 1). Some viral lncRNAs in host cells are not generated from canonical working flow, instead they are processed by unique maturation steps and even degradation of host cellular machineries. For example, flavivirus genome RNA is degraded by 5′-3′ exonuclease Xrn1 in host cells as a defense mechanism. However, flavivirus has developed a special secondary or tertiary structure of its RNA to halt Xrn1 processing to the 3′ end. So Xrn1 stalls on this structures and creates a large amount of degradation intermediates in host cells, named subgenomic flavivirus RNA (sfRNA) or Xrn1-resistant RNAs (xrRNAs) (43) (Table 1), as a specific feature of flavivirus infection. It has to be underlined that viral RNA amount in host cell is highly correlated with viral infection activity. Some RNAs are only expressed during latency, for example latency-associated transcript (LAT) from herpes simplex virus type 1 (HSV-1), while some are highly expressed during the lytic phase, such as PAN RNA. This trait of expression makes viral RNAs to be potential targets in clinical detection of relative virus infection.

**Table 1:** The expressions, functions and mechanisms of viral lncRNAs in viral infection.

**Sources**	**lncRNA**	**Expression/transcription**	**Functions/mechanisms**	**RNA location**	**References**
HCMV	RNA2.7	Highly expressed at early times of infection	Interacting with complex I to prevent GRIM-19 translocalization to stabilizes the mitochondrial membrane potential, resulting in continued ATP production for virus	Cytoplasm	(29, 30)
EBV	oriPts	Expressed during reactivation from EBV latency origin of replication	Modulating paraspeckle-based innate antiviral immune pathway, global viral lytic gene expression, and viral DNA replication during reactivation.	Nucleus	(31)
Group C enterovirus	RNase L ciRNA	Expressed during infection	A competitive inhibitor of the antiviral endoribonuclease RNase L	Cytoplasm	(32)
Flavivirus	sfRNA	Flavivirus genomic RNA degradation intermediates in Xrn1 processing	Oversaturation of Xrn1 degradation and the RNAi machinery	Cytoplasm	(33, 34)
Dengue virus (DENV-2 PR-2B)	PR-2B sfRNA	One of sfRNA	Binding E3 ubiquitin ligase TRIM25 to prevent its deubiquitinylation to unstabilize RIG-I to decrease IFN production and antiviral responses	Cytoplasm	(35)
Dengue virus (DENV-2)	DENV-2 sfRNA	One of sfRNA	Binding to host RNA-binding proteins to antagonize their function in ISG translation, as a molecular sponge of anti-viral effectors.	Cytoplasm	(36)
Adenovirus	VA RNA	Transcribed by polymerase III	Sequestration of several key members of the RNAi pathway and cytoplasmic sensor PKR.	Cytoplasm	(28, 37)
EBV	EBERs	Transcribed by polymerase III	Binding PKR to prevent its dimerization and auto-phosphorylation and signaling to eIF2a, promoting translation of viral proteins	Cytoplasm	(26, 27)
HIV	ASP RNA	Antisense transcript	Recruiting PRC2 to the HIV-1 5' LTR leading to suppressive H3K27 trimethylation and establishment of HIV-1 latency	Nucleus	(38)
KSHV	PAN RNA	Highly expressed during the lytic phase by polymerase II	Guiding PRC2 to the KSHV genome to mediate activation of viral gene expression to produce infectious virus; interacting with H1/H2A, SSBPs, and IRF4 to decrease the expression of IFNγ, IFNα, IL18, and RNase L	Nucleus	(25, 39–42)

## Mammalian lncRNAs

As RNA polymerase I only transcribes ribosomal RNAs in eukaryotes, most lncRNAs discussed here are transcribed by polymerase II (Table 2) and undergo similar splicing and modification processing as message RNA (mRNA), such as methylguanosine at the 5′-terminus and a polyadenylated tail at the 3′-terminus. These lncRNAs are often referred to as canonical lncRNA. Genetically, compared with mRNA, lncRNAs harbor fewer of exons pre transcripts and alternately spliced isoforms per gene locus, and the lengths of lncRNA transcripts are more concentrated within the range of 100–1000 nucleotides (66). RNA polymerase III also transcribes some regulatory ncRNAs, such as RNA Alu, 7SK, BC200, B1 and B2 RNAs (67). Compared with canonical lncRNA, these regulatory RNAs are shorter in length, usually no more than 500 nucleotides, and function mainly through interacting with transcription factors and RNA polymerase II to regulate transcription (68) or influencing mRNA translation (69).

**Table 2:** The expressions, functions, and mechanisms of host lncRNAs in viral infection.

**lncRNA**	**Expression/transcription**	**Gene locus**	**Functions/mechanisms**	**Location**	**References**
NRON	Highly expressed in CD4+ T lymphocytes	Antisense overlapping with coding gene MVB12B	Retaining transcription factor NFAT in the cytoplasm to suppress NFAT-mediated viral gene activation	Cytoplasm	(44)
lncRNA-ACOD1	Induced in many viral infections	An intergenic lncRNA proximal to ACOD1 gene	Binding GOT2 to promote metabolite production to promote viral replication	Cytoplasm	(45)
7SL RNA	Pol III-transcribed SRP RNA	Multicopy gene	Encapsidated into diverse retroviruses and functions as a key cofactor of the antiviral protein A3G	Cytoplasm	(46, 47)
lnc-Lsm3b	Induced by IFN	Sense overlapping with coding gene LSM3	Binding RIG-I to obstruct its conformational shift, prevented downstream signaling, and terminated IFN production	Cytoplasm	(48)
Lethe	Induced by proinflammatory cytokines via NF-κB or glucocorticoid receptor agonist treatment	Intergenic lncRNA	Binding NF-κB activatory subunit RelA to inhibit RelA DNA binding and target gene activation, as a negative feedback of NF-κB.	Cytoplasm	(49)
PACER	Chromatin factor CTCF establishes an open chromatin domain and induces its expression	Antisense head to head lncRNA with gene COX-2	Binding repressive subunit p50 to occlude it from COX-2 promoter, potentially facilitating interaction with active NF-κB dimers p65/p50 to promote COX2 transcription *in cis*	Nucleus	(50)
lincRNA-Cox2	Induced by TLR ligands through MyD88 and NF-κB.	An intergenic lncRNA proximal to Cox2 gene	Binding with hnRNP A/B and A2/B1 to regulates expression of a group of immune response genes	Nucleus	(51)
THRIL/linc1992	Downregulated by TNFα or TLR activation in viral infection	An intergenic lncRNA	Binding hnRNPL to promote transcription of the TNFα gene by binding to its promoter	Nucleus	(52)
NeST/ IfngAS1	Expressed in T cells by NF-κB, STAT4 and T-bet activation	Antisense overlapping with gene IFNG	Promoting IFNγ expression through binding WDR5 and altering histone 3 methylation at the IFNγ locus	Nucleus	(53–56)
LUARIS/ lncRNA#32	Downregulated by IFN	Antisense overlapping with gene HECW1	Binding hnRNPU to activate ATF2 to promote the expression of multiple ISGs	Nucleus	(57)
EGOT	Induced by IFN, HCV, influenza, and SFV	Antisense overlapping with coding gene ITPR1	Inhibiting multiple ISGs' expression as a negative feedback regulatory mechanism of IFN pathway	–	(58)
NRAV	Downregulated during IAV infection	Antisense overlapping with coding gene DYNLL1	Altering epigenetic histone modifications on the promoters of *IFITM3* and *MxA* to attenuate their initial transcription rates	Nucleus	(59)
NRIR/ lncRNA-CMPK2	Upregulated significantly by IFN	An intergenic lncRNA proximal to CMPK2 gene	Repressing expression of many antiviral ISGs probably through interacting with transcription factors or chromatin.	Nucleus	(60)
BISPR	Induced by IFN	Antisense head to head lncRNA with gene BST2	Promoting BST2 expression *in cis* through obstructing PRC2 at the promoter of BST2 to facilitate its transcription and interacting with EZH2 to overlap with enhancer region	Nucleus	(61)
NEAT1	Increased by HSV-1 and HIV	Intergenic lncRNA	Increasing viral gene expression and viral replication for HSV1; negatively regulating viral production for HIV; promoting RIG-I, DDX60 and IL8 expression by removing inhibitory effecter SFPQ to paraspeckles.	Nucleus	(62–65)

Genomic location of lncRNA usually closely associates with its molecular function or mode of activity. Based on the relationship with the nearest coding gene in genome, host lncRNAs are classified into four categories. LncRNA genes with a distance further than 5-kb to the nearest coding genes are defined as intergenic lncRNAs, which are also called long intergenic noncoding RNAs (lincRNAs). However, 5-kb distance is an arbitrary threshold by experience, sometime it varies case by case. Intergenic lncRNAs are functionally referring to lncRNAs without overlapping or sharing transcriptional machinery with other genes, which tend to be independent genes at both expression and function levels. So they are easier to be genetically manipulated compared with other lncRNAs (70). Intergenic lncRNAs prefer to function through exerting *in trans* activity far from their transcription site, which is the case for Firre affecting topological organization of multichromosomal regions through interacting with the nuclear-matrix factor heterogeneous nuclear ribonucleoprotein U (hnRNPU) (71). Some intergenic lncRNAs also influence the expression of the nearby genes via promoter competition for a shared set of enhancers (72) or via histone modification regulation (73).

**Figure 2:**
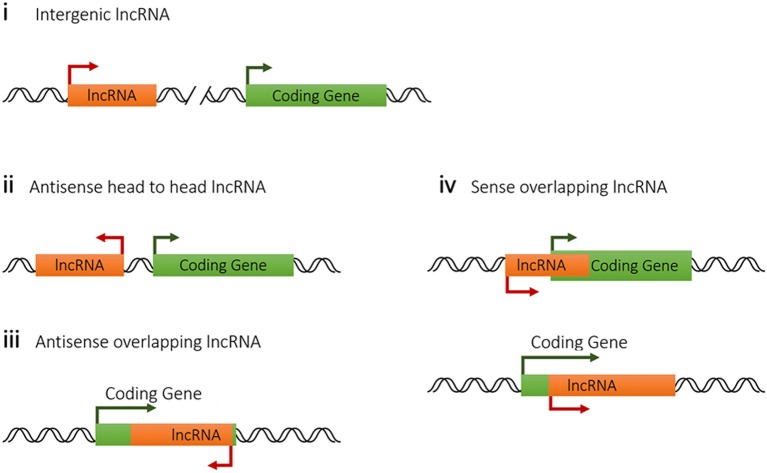
Classification of lncRNAs from host genome according to their positions to the nearest coding genes.

To categorize functionally, lncRNAs with a coding gene in less than 5-kb distance are classified into three biotypes, antisense head to head lncRNA, antisense overlapping lncRNA and sense overlapping lncRNA (Figure 2). The antisense head to head lncRNAs or divergent lncRNAs, which means they are transcribed in the antisense direction and positioned head to head to protein-coding genes, account for a significant proportion of host lncRNA (about 20% in mammals and about 70% in lower metazoans) and are predicted to be strongly related to functions in transcription and development (74). Generally, the expression of antisense lncRNA are likely to be co-expressed with its overlapping coding gene as reported (10), probably because this lncRNA-coding gene pair shares the same chromatin transcriptional loop with a synergistic effect. However, the expressions of sense overlapping lncRNA do not exhibit obvious correlation with coding gene and even in many cases sense lncRNA depresses the expression of proximate coding gene, such as lncRNA Flicr dampening Foxp3 expression in Treg cells (75), probably through competing for shared transcriptional elements. So it might be summarized that lncRNAs tend to promote its nearby antisense strand transcription while depress the sense nearby gene expression.

There are always some unique lncRNA species that resist common categorization, for example intron-derived lncRNA with snoRNA ends (sno-lncRNAs) (76) and exon-derived circular RNAs (circRNAs) (77), which are also transcribed by RNA polymerase II but do not have 5′ and 3′ terminus of mRNA due to RNA splicing alternation. They are believed to function through altering splicing, affecting parental gene expression, or sponging microRNA binding.

## Expression of lncRNA During Viral Infections

Virally derived RNAs are relative simple in their expression regulations, usually constitutively expressed upon invasion or induced expressed after the latency stage, probably due to our little knowledge on it. However, host-derived lncRNAs have been proved to exhibit more complicated spatial and temporal expression patterns, which this part of review focuses on.

A member of high throughout transcriptomic analyses in both human and mouse have revealed that viral infection induced great changes in host cell transcriptome, which includes large amount of protein coding genes along with lncRNAs (78). Promoter prediction and expression correlation analysis revealed that a great proportion of induced lncRNAs in viral infection are direct targets of IFN signaling or IFN-stimulated genes (ISGs). This is supported by experimental data from expression profiling of mouse macrophages with vesicular stomatitis virus (VSV) infection or recombinant IFNβ stimulation (45) and primary human hepatocytes with direct IFNα treatment (60). Interestingly, host lncRNAs have different expression pattern dynamics—some are induced in minutes while some are expressed in days. Another high throughput study in HuH7 hepatocytes that focused on the late time-point of IFN stimulation (72 h post high dose IFNα2 treatment) revealed another group of IFN-regulated lncRNAs, half being upregulated and half downregulated, while nearly all protein encoding genes changed were upregulated (79, 80). This elegant study clearly indicates that host lncRNAs have more complicated roles in gene expression regulation than what we previously expected.

Aside from IFN-regulated lncRNAs, there are some host lncRNAs that are induced by virus as viral hijacked lncRNA, whose expression does not rely on IFN signaling, as revealed by experiment data from wild type and IFN receptor deficiency macrophages (45). These IFN-independent lncRNAs tend to be manipulated by virus and involved in viral invasion. This is in the case of two intergenic lncRNAs, lncRNA-ACOD1 and VIN (virus inducible lincRNA). LncRNA-ACOD1 expression can be induced by many virus, including VSV, Sendai virus (SeV), HSV-1, and vaccinia virus (VACV), partly dependent on NF-κB signaling, and its induction is attenuated by IRF3 signaling as IRF3 knockout led to a higher expression, indicating lncRNA-ACOD1 is a favorable lncRNA for virus rather than host (45). VIN was identified as viral induced lncRNAs in human lung epithelial cells by several influenza A virus (IAV) strains (H1N1, H3N2, H7N7) and VSV. However, it could not be induced by infection of influenza B virus, treatment of RNA mimics stimulus, or treatment of IFNβ, indicating VIN was selectively utilized by some virus during millions of years' evolution (81). Interestingly, there are some host RNAs identified to be regulated by only one specific virus, such as CSR19, CSR21, CSR26, and CSR34 in hepatitis C virus (HCV) infection (58), indicating their specific role for HCV infection.

It is noteworthy that some host lncRNA's expression also is responsive to viral infection in some specific organs, for example placenta in mammal, which is usually believed to an immune tolerance place. A recent study pointed out that SeV infection of human trophectoderm progenitor cells induced an lncRNA expression, named lncRHOXF1, which was transcribed from the X chromosome. lncRHOXF1 promoted the host response to viral infections (82).

## lncRNA Working Mechanisms at the Molecular Level

As for the working mode of RNA, aside from riboswitches and ribozymes whose RNA structure alone performs the functional units, most ncRNAs operate as complexes with proteins, such as ribosome, telomerase, snRNP, snoRNP, and RISC complex of microRNAs. LncRNAs also perform in a similar manner, interacting with diverse proteins to perform different functions.

In cell nucleus, lncRNAs usually associate with chromatin modification protein or epigenetic modulator to regulate coding gene expression *in trans* or *in cis*, for example host lncRNA-EPS and lncRNA-COX1 (51, 83). In some cases, the activity of one lncRNA could have more than one target or even the whole chromatin. LncRNA Firre was reported to interact with multiple sites of the genome and influences chromatin topological organization through interacting with the nuclear-matrix factor hnRNPU (71). The well-known lncRNA XIST from X chromosome regulated the expression status of the whole X chromosome through recruitment of the polycomb repressive complexes PRC1/2 (84). Another example is NORAD, a conserved and broadly expressed long noncoding RNA, which preserves the whole genome stability in mitosis by serving as a molecular decoy for PUMILIO proteins (85).

Many lncRNAs utilize base pairing to bind other molecules of nucleic acids, such as microRNAs, as competing endogenous RNAs (ceRNAs) to regulate other RNA transcripts (86, 87). For instance, lncRNA linc-MD1 binds miR-133 and miR-135 in myocytes to liberate the expression of muscle-specific transcription factors MAML1 and MEF2C (88). LncRNAs also function through associating signaling transductors or enzymes. In the cytoplasm of dendritic cells, host lncRNA lnc-DC binds transcription factor STAT3 to protect its phosphorylation on tyrosine-705 through preventing protein phosphatase SHP1 binding (89). Another example is from Song's lab, showing that NF-κB-upregulated lncRNA NKILA binds to NF-κB/IκB complex and directly masks phosphorylation motifs of IκB, thereby inhibiting IKK-induced IκB phosphorylation and thus NF-κB activation (90).

**Figure 3:**
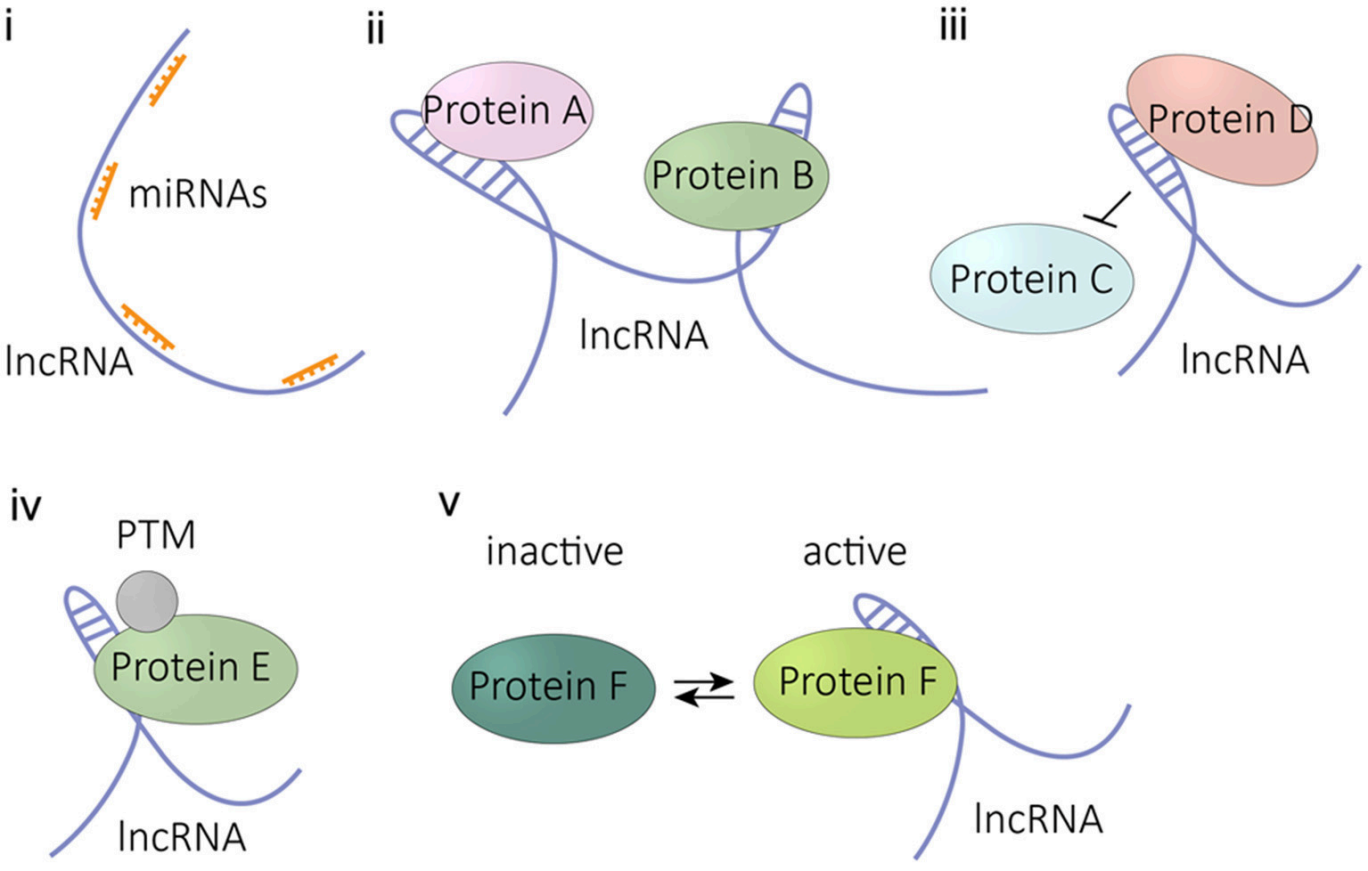
Molecular modes of lncRNA activities. (i) competitive binding microRNA as a sponge RNA, (ii) binding protein as a competitive inhibitor, (iii) binding one protein to prevent its interaction with another protein, (iv) influencing the PTM modification of the binding protein, (v) affecting the activity of the binding protein. PTM, post translational modification.

In summary, lncRNAs associate with various molecules through base-pairing or spatial structure interaction to exploit different actions as illustrated in Figure 3. However, it is still unknown whether there are other undiscovered modes of action for lncRNA.

## lncRNAs Regulating Viral Life Cycle

The course of viral infection cycle in host cells is the trigger of host immune defense and the causes of pathological damages. Taking enveloped virus for example, the life cycle of virus can be summarized as following: First, virus enters the host cell membrane through receptor-mediated endocytosis, followed by viral genome release into the cytoplasm. After passing through a refined procedure of genome integration and latency, or without latency, viral genome utilizes cellular machinery and viral enzymes to synthesize protein component, replicate their genome, and then assemble new progeny virions. Finally infective virions are released and infect other host cells. While large amount of molecules and medicines have been proved to target the process of viral life cycle, increasing number of lncRNAs are revealed to regulate different steps in this process.

### Viral Gene Expression

During HSV infection, host paraspeckle lncRNA NEAT1, together with paraspeckle protein P54nrb and PSPC1, associates with HSV-1 genomic DNA and recruits STAT3 to paraspeckle. They facilitate the interaction between STAT3 and viral gene promoters to increase viral gene expression and viral replication, as reported in both human and mouse cell lines (62). However, during human immunodeficiency virus (HIV) infection, NEAT1 and paraspeckle bodies negatively regulate viral production in human cell line through increasing nucleus-to-cytoplasm export of instability element (INS)-containing HIV-1 mRNAs to promote HIV-1 transcript splicing (63). These results indicate that NEAT1 and paraspeckle probably perform different roles in different viral infections. As an important nuclear body for gene expression regulation, paraspeckles are targeted by some viral RNAs. Epstein-Barr virus (EBV) derived lncRNAs oriPtL and oriPtR are bi-directionally transcribed RNAs from EBV latency origin of replication in the nucleus. They bind paraspeckle protein NONO and RNA-editing enzyme ADAR to modulate global viral lytic gene expression and viral DNA replication through an evolutionarily conserved and thermodynamically stable hairpin at their family of repeat (FR) regions (31).

### Viral RNA Stability

To affect viral RNA stability or directly degrade viral RNA during infection, host cells have developed many defense mechanisms, including endoribonuclease RNaseL, exoribonuclease Xrn1, and RNAi pathway. However, some viruses have evolved their own strategies to counteract this degradation, such as poliovirus and other group C enterovirus. RNaseL, which degrades single stranded RNAs, is activated by the second messenger 2′-5′-linked oligoadenylate (2–5As) produced by its synthetase OAS, another important sensor for dsRNA in the cytoplasm. Poliovirus and other group C enterovirus have a conserved RNA structure within the open reading frame which functions as a competitive inhibitor of the antiviral endoribonuclease RNaseL (Figure 1). Hence, this viral RNA was named the RNaseL competitive inhibitor RNA (RNase L ciRNA) (32). Another example is flavivirus sfRNA. As described in the sections above, flavivirus produces large amount of sfRNAs which can halted exoribonuclease Xrn1 digesting, and sfRNAs can also repress the RNAi machinery by saturation of endoribonuclease Dicer (33, 34). It is the same for adenovirus VA RNAs whose structure and high abundancy sequestrate several key members of the RNAi pathway, such as Dicer and Exportin 5 (37).

### Transition and Rhythm of Viral Life Cycle

Stage transition and rhythm of viral life cycle is closely associated with the course of pathological processing and immune response status. So maintaining viral latency or switching to lytic reactivation is intensively regulated by both virus and host, which involves many lncRNAs from both sides. It has been reported that viral RNA ASP, an HIV antisense transcript, recruits polycomb repression complex 2 (PRC2) to the HIV-1 5′ LTR, resulting in the accumulation of suppressive H3K27 trimethylation to facilitate the establishment of HIV latency (38). Another case is PAN RNA from KSHV. During initiation of the lytic phase, KSHV expresses a highly abundant long noncoding transcript, viral PAN RNA, which guides specific demethylases and PRC2 to the KSHV genome to mediate activation of viral gene expression, leading to the production of infectious virus and lytic infection (39–41). On the other hand, host cells are also trying to influence the life cycle of virus. It has been reported that host lncRNA NRON, highly expressed in resting CD4^+^ T lymphocytes, maintains HIV-1 latency by retaining transcription factor NFAT in the cytoplasm to suppress NFAT-mediated viral gene activation (44).

### Metabolic Regulation

As viral replication requires large amounts of material and energy, it has developed some strategies to hijack metabolic network of host cells to direct metabolite flow to their benefit. However, for a long time we knew little about the underlying molecular mechanism of how virus performs. Recently, a host intergenic lncRNA, lncRNA-ACOD1, was reported to be induced by many viruses as mentioned above and further results from our lab revealed that it promoted viral infection through manipulating host cell metabolism. In the cytoplasm, it directly binds to glutamic-oxaloacetic transaminase 2 (GOT2) near the substrate niche. This RNA-protein interaction promotes the catalytic activity of GOT2 to facilitate metabolite production, such as L-aspartate, α-ketoglutarate and subsequent lipid production, which feeds virus and accelerates viral replication (45). This work marked lncRNA-AOCD1 as a metabolic regulator that is hijacked by virus in infection. The large number of unstudied virally induced transcripts makes it highly likely that future studies will reveal a much greater share for this class of lncRNAs in regulation of viral infection.

Recently more and more studies have revealed that metabolic regulation is vital for host immune regulation. Metabolite is not just the source of energy and nutrition, but also a regulator of host cells immunity, which is the case for itaconate pathway in innate immunity (91) and tetrahydrobiopterin (BH4) in adaptive immunity (92). Although it still at the beginning, metabolic lncRNA probable also has other functions in immune regulation.

It is noteworthy that while lncRNA-ACOD was revealed to be induced in many organs and tissues, including liver, spleen, lung, and lymph nodes, it has a constitutive high-expression level in heart, which is a high energy-consuming organ. Considering lncRNA-ACOD1 as a metabolic regulator, its high expression in heart indicates that it may also participate in the regulation of cardiovascular function.

If host cells cannot take back the control of metabolism, as a protective mechanism for host cells, initiating mitochondria-induced cell death of viral infected cell at early stage of infection can shut down the energy source for virus and restrict viral infection spreading. However, some viruses have evolved to maintain host cell alive at least until they completely fulfill the infective cycle and infect other cells. John H. Sinclair et al. found a 2.7-kilobase viral RNA transcript (RNA2.7 or Beta2.7) from human cytomegalovirus (HCMV) protected host cells from apoptosis, and RNA2.7 accounts for >20% of total viral transcription at the early stage of infection (12 to 24 h) (29). RNA2.7 interacted with complex I and prevented translocation of its essential subunit GRIM-19 to stabilize the mitochondrial membrane potential, resulting in continued adenosine triphosphate (ATP) production to support virus replication (30).

These two studies above represent delicate strategies in modulating the metabolic viability of the infected cell by noncoding RNAs from both virus and host. Researchers have begun to apply these RNAs or the functional fragment in the diagnoses and therapies of clinical relevant disorders. For example, the 800 nucleotide subdomain of RNA2.7, which plays an anti-apoptotic role and maintains a high level of ATP production in neurons, has been exploited in the development of a novel therapeutic for Parkinson's disease (93).

### Packaging and Releasing

Packaging and releasing is the last step of life cycle for virus, which is also an excellent chance for host to block infection. It has been reported that cytidine deaminase APOBEC3G (A3G) has a broad antiviral activity against diverse retroviruses and retrotransposons, through inducing C-to-U mutations in the minus-strand viral DNA after its encapsidation into virions (94, 95). However, this process relies on polymerase III-transcribed host 7SL RNA, which is the RNA component of SRP, also known as 4.5S RNA. Studies with HIV-1 infection revealed that A3G selectively interacts with 7SL RNA, then interacted with the RNA-binding domain of HIV-1 Gag protein and was preferentially packaged into virus particles. So 7SL RNA encapsulated into retroviruses functions as a key cofactor of the antiviral protein A3G (46, 47), which proposes a new working model for Pol III-transcribed host noncoding RNAs to participate antiviral immune responses.

## lncRNAs Regulating Antiviral Innate Immune Responses

Effective induction of IFN and cytokines along with the downstream effectors expression are crucial for host antiviral response and is known to be orchestrated by multiple mechanisms (96, 97). Nowadays increasing evidences point to the presence of lncRNA-mediated regulatory mechanisms on this pathway. Just like protein regulators, some lncRNAs involved in this mechanism promote this response as positive regulator to strengthen antiviral defense, while others function to attenuate immune responses as a negative feedback regulator to avoid excessive immune pathological effect or are utilized by virus to escape antiviral defense.

### Antiviral Innate Immune Signaling and Cytokine Productions

The first step of host antiviral responses is sensing viral invasion. Interestingly, some viral sensors in host are also regulated by lncRNAs, which is the case of canonical sensor RIG-I. A recent research revealed an IFN-induced host lncRNA lnc-Lsm3b competed with viral RNAs in binding RIG-I monomer. The binding of lnc-Lsm3b to RIG-I protein obstructs the conformational shift of RIG-I protein which is essential for its activation. So lnc-Lsm3b prevents downstream signaling and thereby terminates IFN production. As host lnc-Lsm3b is an immune induced gene at the late stage of innate response, so in viral infection it functions as a negative feedback regulator of RIG-I pathway (48) (Figure 1). Another example comes from viral RNAs, a particular sfRNA from dengue virus DENV-2 clade (PR-2B). It has been reported to bind E3 ubiquitin ligase TRIM25. This binding prevents the deubiquitinylation of TRIM25 by ubiquitin-specific peptidase 15 (USP15). Ubiquitinated TRIM25 is unable to polyubiquitinate RIG-I and stabilize RIG-I, resulting in a significant decrease in the IFN production and an impaired antiviral responses to facilitate viral infection (35) (Figure 1).

Another viral sensor, double-stranded RNA-dependent protein kinase (PKR) is a cytoplasmic sensor of viral RNA, whose activation induces translation inhibition to suppress viral protein synthesis through phosphorylation of eIF2a. However, some viral lncRNAs, such as VA RNAs and EBERs, bind to PKR, but do not induce PKR activation. Instead, they prevent PKR dimerization and auto-phosphorylation. Therefore, signaling through PKR to eIF2a is blocked and translation of viral proteins is properly initiated (27, 28) (Figure 1).

The downstream immune signaling is also regulated directly by lncRNAs through RNA-protein interactions. For example, host lncRNA lnc-DC regulates STAT3 signaling as that is described above. Another example is NF-κB, which is believed to be a key director of inflammatory cytokine expression and late stage IFN production in antiviral immune responses. Lethe, an intergenic lncRNA that is also considered as a pseudogene, is selectively induced by inflammatory cytokines and glucocorticoid receptor agonist. Functionally, it interacts with NF-κB active subunit RelA to inhibit RelA DNA binding and target gene activation, as a negative feedback to NF-κB (49). So Lethe could have profound effect in immune responses. Another example for NF-κB is p50-associated COX2 extragenic RNA (PACER), which is an antisense head to head lncRNA with coding gene cyclooxygenase 2 (COX2). PACER associates with p50, a repressive subunit of NF-κB, and occludes it from the COX-2 promoter to facilitate active NF-κB dimer p65/p50 to COX2 promoter to promote its expression (50). Interestingly, there is an intergenic lncRNA nearby Cox2 (Ptgs2) gene in mouse, named lincRNA-Cox2. However, lincRNA-Cox2 does not alter Cox2 (Ptgs2) expression in mouse. As an immune-induced gene, it regulates expression of a group of immune response genes, including chemokines, chemokine receptors, and ISGs, through binding hnRNP A/B and A2/B1 (51).

Some lncRNA can directly regulate the transcription of cytokines. Inflammatory cytokine tumor necrosis factor α (TNFα) is a potent activator of host immune responses to viral infections. It has been reported its transcription is regulated by an intergenic lncRNA from host genome, named THRIL which is short for TNFα and hnRNPL related immunoregulatory lincRNA. THRIL functions through binding hnRNPL to form a functional lncRNA/hnRNPL complex that binds to TNFα promoter. However, it is downregulated by TNFα or TLR stimulation as a negative feedback mechanism during viral infection (52). As an essential host lncRNA for the formation of nuclear body paraspeckle, NEAT1 regulates many immune-related genes expression, including antiviral cytokine IL8 (64) and host sensor RIG-I and DDX60 (65). NEAT1 transits the splicing factor proline and glutamine-rich protein (SFPQ) to paraspeckle to remove its transcriptional inhibitory effects, promoting the expression of immune responses genes.

Virally encoded RNAs also participate in regulating host IFN and cytokine production as an immune modulator for the sake of virus. KSHV derived PAN RNA was proved in to interact with histone H1/H2A, single-stranded binding proteins (SSBPs) and interferon regulatory factor 4 (IRF4) in infected cells to decrease the expression of IFNγ, IFNα, interleukin-18, and RNase L to facilitate viral infection in primate cell lines (42).

Taken together, despite only small fraction of lncRNAs being studied, existing data points a critical role for this class of lncRNAs in regulation of immune signaling and cytokine expression. Increasing evidence of lncRNA directly interacting with signaling molecules has also been found not only in immune signaling but also in other biomedical flied including p53 signaling (98) and EGFR signaling (99). However, we still do not know how many other sensors, adaptors and transcription factors could interact with lncRNA to be modulated. We just put up some of the remaining questions in antiviral signaling as illustrated in Figure 1: Is TBK1-IRF3 activity regulated by some lncRNA? Does lncRNA interact with signaling adaptors, such as STING or TRAF6, to modulate their protein modifications and functions? Resolving these questions will help us deepen our understanding of relationship between lncRNAs and signaling cascades. Furthermore, as many protein factors have been identified as negative or positive regulators of immune signaling, defining the interaction of the regulatory lncRNAs with these proteins in the context of immune responses will yield an uncharted regulatory network of immune cascades.

### Expression of Interferon-Stimulated Genes (ISGs)

ISGs comprise antiviral effectors and immune regulators and a number of lncRNAs exert their functions through regulating ISGs to modulate the antiviral effect. There are many such cases for IFN-induced lncRNAs. LncRNA NRIR (negative regulator of IFN response) was identified in primary human hepatocytes as an intergenic lncRNA (60), originated from a locus downstream of the protein-coding ISG CMPK2, and therefore it was also named lncRNA-CMPK2. NRIR could be induced by IFNα, IFNβ, and IFNγ through JAK-STAT pathway to generate a spliced polyadenylated nuclear transcript. Functionally, knocking down NRIR in human hepatocyte cell line significantly increased many antiviral ISGs expression, including CMPK2, viperin, IFIT1, IFIT3, ISG15, MxA, CXCL10, and IFITM1, and subsequently decreased HCV replication, suggesting NRIR acts as a repressor of ISGs expression (60). Although its molecular mechanism yet to be further determined, the case of NRIR indicates that lncRNAs play an important role in the feedback loop of IFN-induced gene expression regulation. In addition, NRIR is reported to have a remarkably high level in livers of patients with HCV infection than that in healthy donors, indicating this negative regulator could be utilized by HCV *in vivo* (60).

Another example is host lncRNA EGOT (Eosinophil Granule Ontogeny Transcript), which is a polyadenylated nuclear conserved lncRNA (100). EGOT was first described to be expressed in eosinophils and is thought to function in mature eosinophils (101). Later studies from GTEx Consortium revealed that the highest levels of EGOT were found in nonhematopoietic tissues such as breast, pancreas, pituitary, vagina and kidney cortex (102). Interestingly, EGOT genomic locus in human being was marked by monomethylation of Lys4 of histone H3 (H3K4), but not trimethylation of H3K4, indicating that EGOT could be an enhancer RNA (103). Its function remained obscure until a recent study using human liver cell line HuH7 cells revealed that the level of EGOT was dramatically induced by viral infection, such as HCV, influenza, and Semliki Forest virus (SFV), and high doses of IFNα stimulation (58), and furthermore, knockdown experiment in HCV infected cells revealed that EGOT negatively regulated antiviral responses through inhibiting a subset of ISGs' expression as a negative feedback regulatory mechanism of IFN pathway. However, this mechanism is often utilized by viruses, such as HCV, influenza, and Semliki Forest virus (SFV) (58).

While described above are two lncRNAs upregulated in viral infection, host lncRNA NRAV (Negative Regulator of Antiviral Response) was markedly reduced in infection with influenza virus and a number of other viruses in several cell lines. NRAV was firstly described in a study on genes expression changes in response to influenza virus H1N1 infection in human alveolar epithelium cell line A549 (59). Importantly, overexpression experiments with cDNA microarray analysis revealed NRAV depressed the expression of many antiviral effecters, including IFIT2, IFIT3, IFITM3, MxA, and OASL (59). Studies in human cell lines or transgenic mice showed that enforced expression of NRAV markedly promoted viral replication while knockdown of NRAV suppresses viral replication in IAV infection model. Furthermore, molecular studies revealed that the spatial structure of NRAV associates with the promoters of *IFITM3* and *MxA* to alter their epigenetic histone modifications to suppress their initial transcription rates (59). It is noteworthy that NRAV is an antisense overlapping lncRNA and locates in the first intron of *DYNLL1* gene encoding human dynein light chain, however, they are transcribed as independent operating unit, which is a unique working model *in trans* for antisense overlapping lncRNA.

Host lncRNA LUARIS (lncRNA up-regulator of antiviral response IFN signaling) is also down-regulated, like NRAV in viral infection, and as it was named, it functions to promote ISGs expression. LUARIS was identified in a screen for IRF3-dependent genes in HuS immortalized human hepatocytes as an IFN-reduced lncRNA originally named lncRNA#32 (57). It was reported that during HBV or HCV infection in primary hepatocytes, LUARIS associates with hnRNPU and functions through activating transcription factor 2 (ATF2) to promote the expression of multiple ISGs. And silencing of LUARIS dramatically reduced the level of ISGs' expression and increased cellular sensitivity to encephalomyocarditis virus (EMCV) infection (57). These data indicate LUARIS has evolved to control the magnitude of the IFN response through multiple regulatory pathways to prevent possible toxicity of overstimulation.

While many lncRNAs regulate the expressions of multiple ISGs, some lncRNAs can only modulate a single ISG target, for example BISPR (BST2 IFN-Stimulated Positive Regulator). Host lncRNA BISPR belongs to antisense head to head lncRNAs, which is transcribed from a bidirectional promoter shared with BST2/Tetherin. BST2 is an IFN-induced restriction factor that blocks the budding of enveloped viruses by tethering them to the cell surface. Independent studies from two groups revealed that BISPR/BST2 gene-pair is induced by IFN stimulation in many cells, such as human hepatocyte cell line HuH7 (79, 80) and monocyte cell line THP1 (61). Interestingly, the increase of BISPR expression precedes that of BST2 after IFN stimulation, indicating that BISPR induces or facilitates the initiation of BST2 transcription. Future studies revealed that BISPR knockdown reduced BST2 expression and ectopic expression of BISPR RNA enhanced BST2 expression, indicating that BISPR RNA mediates this function, rather than the transcription (61). Mechanistically, BISPR obstructs the repressive activity of PRC2 at the promoter of BST2 to facilitate the transcription. BISPR also interacts with methyltransferase component EZH2 and an enhancer region to promote the formation of enhancer-promoter complex. Since many lncRNAs belong to antisense head to head lncRNAs and many immunity-related genes have bidirectional promoters, the mechanism of BISPR and BST2 study shed light on these antisense lncRNA family's functional and molecular exploration.

Aside from transcription, the translation of ISG mRNA is also regulated by noncoding RNAs. It was reported that conserved host RNA-binding proteins G3BP1, G3BP2, and CAPRIN1, which were required for ISG translation, were targeted by a non-coding RNA from dengue virus. Human pathogen dengue virus is a positive-strand RNA flavivirus and it produces abundant non-coding sfRNA, which directly binds to G3BP1, G3BP2, and CAPRI, as a molecular sponge, to antagonize ISGs translation (36). This mechanism impairs establishment of the antiviral defense of host cells, allowing virus to replicate and escape from the IFN response. Interestingly, Dengue sfRNA response has not been observed in other flaviviruses. The unique association of this sfRNA molecule to dengue viral pathogenesis provides a potential molecular target for clinical diagnosis and future therapeutic options for dengue virus infection.

## lncRNAs in Antiviral Adaptive Immunity

lncRNA transcriptome profiling in different T cell lineages has been performed and characterized in both humans and mice (104, 105). Thousands of lncRNAs have been identified to closely associate with T lymphocyte differentiation and a number of them are identified as novel T helper (Th) cell lineage-specific lncRNAs. Most of these lncRNAs are intragenic or adjacent to lineage-specific protein-coding genes in the genome. And many were bound and regulated by the key transcription factors T-bet, GATA-3, STAT4, and STAT6 as revealed by RNA-Seq data and ChIP-Seq data (106). Some of these lncRNAs have been proved to have functions for T cells, including an enhancer-like lncRNA called IfngAS1 (also known as Tmevpg1 or NeST) promoting Th1 cytokine IFNγ expression (53), host lncRNA Th2-LCR lncRNA controlling Th2 cytokines IL-4, IL-5, and IL-13 expression (107), and lincR-Ccr2-5′AS regulating the migration of Th2 cells (106). However, few of them have been characterized for molecule mechanism. To our knowledge, it is the only case for IfngAS1 located adjacent to the *IFNG* in both mice and humans (54, 55). IfngAS1 promotes IFNγ expression by binding to WD repeat-containing protein 5 (WDR5), a component of histone H3 lysine 4 (H3K4) methyltransferase complex, and alter histone 3 methylation at the IFNγ locus (53, 56).

Despite these discovery in T lymphocytes have been achieved, many questions are yet to be answered. For example, it is not clear whether T cell activation signaling is regulated by lncRNA as an intrinsic regulation; whether TCR complex involves the interaction with lncRNA during T cell activation and whether B lymphocyte differentiation and function being regulated by lncRNA. The lack of mechanistic insight in this field is due, in part to technical obstacles. For example, cell-specific gene-manipulating for lncRNA genes *in vivo* is much harder than coding genes, because base-pair insertions usually do not lead to functional mutation for noncoding genes. The development of gene editing techniques, such as CRISPR system will provide more convenient approaches for research to manipulate lncRNA genes in specific lymphocytes in the future. Other obstacles include the low abundance of samples, as the number of one specific T cell subtype is very low. With the development of trace amount detection technique, such as SHERLOCK (108) and DETECTR (109), and super-resolution structured illumination microscopy (SIM)^2^ and Cryo-scanning electron microscopy (Cryo-SEM), RNA molecule will be better detected for small amount or even in single cell.

## Challenges and Perspective

lncRNAs from host and virus exert their functions through various mechanisms, such as associating with transcription factors, chromatin modifiers, signaling adapters, enzymes and microRNAs, to influence gene expression, host metabolism, post translational modification, and protein activities. Although great achievements have been obtained in the field of lncRNA, there are still a significant number of concerns to be solved. While the linear sequence of RNA is relative easy to analyze, RNA spatial structure is still difficult to be examined or predicted as the RNA structure is flexible and usually interacts with other molecules *in vivo*. Nevertheless, great effort has been made to interpret the physiological structures of RNAs using elaborate biochemical methodologies to distinguish single-strand, double-strand, exposed, and buried regions. Some approaches are used to resolve one specific lncRNA structure at a single-nucleotide resolution (110) and some are designed to reveal the whole higher-order transcriptome structure in living cells (111–113). Nevertheless, RNA modification adds another layer of complexity in RNA structural and functional research. One example is that a study from Tao Pan's laboratory demonstrated that RNA local structures are altered by one site of *N*^6^-methyladenosine (m^6^A), which is the most abundant modification in eukaryotic RNA (114, 115). This local change in structure increases the binding by heterogeneous nuclear ribonucleoprotein C (hnRNPC) (116). So the RNA functional studies have evolved to the combination of linear sequence, spatial structure and RNA modification, which set a higher level of requirement for experimental exploration and bioinformatics analysis.

Another challenge in RNA mechanistic study is the identification of lncRNA binding molecules. Aside from nucleic acid, protein is so far the only recognized molecule that RNA interacts with. Whether there are other compounds, such as small chemical molecules or metal ions, interacting with lncRNA *in vivo* is largely unknown. Nevertheless, a number of approaches have been developed to get comprehensive profile of RNA-binding proteins (RBPs) *in vivo*. Ultraviolet (UV) crosslinking has been used to covalently stabilize native protein-RNA interactions in living cells. The crosslinked proteins are isolated by oligo (dT) purification for mass spectrometry identification. This approach, named RNA-protein interactome capture, identified over a thousand RBPs within different cells and species, such as human HeLa and HEK293 cells (117, 118), mouse embryonic stem cells (119), Saccharomyces cerevisiae (120), and Caenorhabditis elegans (121). Many proteins identified were not previously recognized to bind RNA, namely unorthodox RBPs, include many metabolic enzymes, regulators of alternative splicing, the E3 ubiquitin ligase, and the FAST kinase domain-containing protein 2 (FASTKD2) (122, 123). Furthermore, to determine how RBPs bind to RNA in living cells, Matthias W. Hentze et al. have improved the resolution of this approach from protein level to RNA binding peptide level, by adding a protease digestion step followed by a second round of oligo (dT) capture and mass spectrometry. They have discovered numerous RNA-binding domains (RBDs) in human HeLa cells and revealed that catalytic centers and protein-protein interacting domains are preferred RNA binding sites (124). Interestingly, nearly half of the RNA-binding sites were mapped to intrinsically disordered protein regions, indicating flexible protein domains are the favorable part for RNA-protein interactions. Recently, a new approach, RNA interactome with click chemistry (RICK), has been developed to capture the nascent RNA-protein interactome. Using this method, newly transcribed RNAs were integrated with 5-ethynyluridine and after UV crosslinking, the RNA-protein complex was labeled with biotin via click chemical reaction and subjected to purification and identification (125). This method allows identification of proteins bound to a wide range of RNA species, including the nonpolyadenylated RNAs that were neglected in the past.

Many techniques are designed to examine the binding proteins of one specific RNA molecule (126). Ci Chu et al. developed the method of chromatin isolation by RNA purification (ChIRP) using antisense DNA oligonucleotides to capture and purify specific lncRNA-chromatin complexes, initially to address lncRNA-binding sites on the genome (127). They further developed the methodology that enabled the identification of lncRNA-binding proteins and RNA-RNA interactions (128, 129). Two other groups have also developed similar approaches independently, RNA antisense purification (RAP) (130) and capture hybridization analysis of RNA targets (Chart) (131), with some differences in cross-linking and chromatin shearing. As ChIRP-like methods use chemical cross-linking, they do not differentiate direct interactions from indirect interactions. Another method named cross-linking ligation and sequencing of hybrids (CLASH) uses UV crosslinking that captures only direct RNA-protein interaction, which is suitable for investigation of RNA-protein interaction in nucleus (132).

Some lncRNAs are not sensitive to siRNA mediated RNA degradation, especially the ones located in the nucleus, as siRNA machinery is mainly located in cytoplasm. So knocking down and knocking out strategies often interfere lncRNA studies. Despite the technical hurdles and difficulties, much effort has been made to inactivate lncRNA genes in mouse models and these studies have made great discoveries. An elegant review by Lingjie Li et al. summarized genetic targeting strategies used to study lncRNA loci *in vivo* (133). However, these gene deletion approaches are difficult to scale up for genome-wide functional screening of lncRNAs, as many lncRNA are induced or suppressed in viral or other immune relative models. Happily, integrated genome-wide CRISPR interference (CRISPRi) and CRISPR activation (GRISPRa), a novel systematic functional screening system of lncRNAs has been developed (134). This technique represses or activates transcription via recruitment of a nuclease-dead Cas9 (dCas9) enzyme to the transcriptional start site (TSS) of genes by a customizable single guide RNA (sgRNA). A study designed a sgRNA library targeting 16,401 lncRNA loci, with 10 sgRNAs against each lncRNA TSS. In seven diverse human cell lines, 499 lncRNA loci were identified to be necessary for robust cellular growth. Surprisingly, the majority of these lncRNA genes showed growth modifying function exclusively in one cell type, and not a single lncRNA modified cell growth across all the cell line tested (135). This is a promising approach for lncRNA functional screening in other models, for example it could be used for the identification of viral specific lncRNAs or cell type-specific antiviral lncRNAs.

As illustrated by the literature, the most significant character that lncRNAs differ from other RNA molecules is that lncRNAs exhibit high cellular- and organ- specific expression patterns. Therefore, lncRNAs modulate protein activities and signaling pathways only in specific cell types. Understanding the mechanisms of specific expression regulation at multiple levels, including but not limited to specific transcriptional factors, epigenetic modification, and local chromatin spatial organization, will help us better understand spatial and temporal regulation of lncRNAs and choose more specific interfering target in certain pathological circumstances, such as viral infection. However, as the strong correlation between lncRNA expression patterns and its functions, analysis of lncRNA expression profiles in different cells under different physiological and pathological models is a good way to predict the relevant functions of one specific lncRNA or a panel of lncRNAs. Furthermore, combining more omics data at the same time, such as transcriptome, epigenomics, proteinomics, phosphoproteomics and metabolomics, and conducting integrated correlation analysis with coding genes, epigenetic modification, protein modification and metabolite, will provide more detailed function information of lncRNAs and might draw the whole regulatory network draft for us.

## Author Contributions

The author confirms being the sole contributor of this work and has approved it for publication.

### Conflict of Interest Statement

The author declares that the research was conducted in the absence of any commercial or financial relationships that could be construed as a potential conflict of interest.
